# Effects of Carbonization Parameters of Moso-Bamboo-Based Porous Charcoal on Capturing Carbon Dioxide

**DOI:** 10.1155/2014/937867

**Published:** 2014-08-13

**Authors:** Pei-Hsing Huang, Jhih-Wei Jhan, Yi-Ming Cheng, Hau-Hsein Cheng

**Affiliations:** Department of Mechanical Engineering, National Pingtung University of Science and Technology, Pingtung 912, Taiwan

## Abstract

This study experimentally analyzed the carbon dioxide adsorption capacity of Moso-bamboo- (Phyllostachys edulis-) based porous charcoal. The porous charcoal was prepared at various carbonization temperatures and ground into powders with 60, 100, and 170 meshes, respectively. In order to understand the adsorption characteristics of porous charcoal, its fundamental properties, namely, charcoal yield, ash content, pH value, Brunauer-Emmett-Teller (BET) surface area, iodine number, pore volume, and powder size, were analyzed. The results show that when the carbonization temperature was increased, the charcoal yield decreased and the pH value increased. Moreover, the bamboo carbonized at a temperature of 1000^°^C for 2 h had the highest iodine sorption value and BET surface area. In the experiments, charcoal powders prepared at various carbonization temperatures were used to adsorb 1.854% CO_2_ for 120 h. The results show that the bamboo charcoal carbonized at 1000^°^C and ground with a 170 mesh had the best adsorption capacity, significantly decreasing the CO_2_ concentration to 0.836%. At room temperature and atmospheric pressure, the Moso-bamboo-based porous charcoal exhibited much better CO_2_ adsorption capacity compared to that of commercially available 350-mesh activated carbon.

## 1. Introduction

The concentration of greenhouse gases, including carbon dioxide, methane, nitrous oxide, chlorofluorocarbon, and ozone, in the atmosphere is increasing [1–8]. The greenhouse effect leads to global warming, sea level rise, and ecosystem imbalance, threatening the existence of many organisms. Therefore, the development of technology for the adsorption, separation, and capture of various greenhouse gases is an urgent issue. The increase in CO_2_ is mainly from the combustion of fossil fuels, which is generally recognized as the major cause of global warming [1]. At present, fossil fuels such as coal, oil, and natural gas account for 85% of the global energy consumed. These fuels account for over 40% of the total CO_2_ emissions [6]. CO_2_ is an innocuous, inexpensive, and renewable gas, which can be used to replace many toxic organic solvents, chemicals, and intermedia [6]. Capturing CO_2_ can effectively reduce CO_2_ emission, as well as creating potential economic benefits.

The main considerations for capture methods are the construction cost of trapping equipment and the energy consumption. CO_2_ trapping methods commonly involve adsorption using chemical/physical adsorbents, cryocondensation, and membrane separation. Adsorption using various adsorbents (e.g., CaCO_3_/CaO compounds, carbon-based porous materials, and mesoporous silica-based particles) was identified as a potential technology by the Intergovernmental Panel on Climate Change (IPCC) [1, 6]. These materials have their advantages and drawbacks in capturing carbon dioxide. Combining their particular advantages is helpful toward developing novel adsorbing materials.

Porous carbon-based materials have high thermal and chemical stability and excellent adsorption capacity [1–8]. Their low cost and recoverability give them potential as pollution prevention materials. Tamon et al. [2] used activated carbon impregnated with metal halide to adsorb CO and measured the adsorption isotherm. The results showed that the impregnated activated carbon had a well adsorption capacity. Wu et al. [3] discussed the adsorption capacities of bamboo charcoal and activated carbon with micropore, mesopore, and macropore distributions based on different functional groups of bamboo charcoal and activated carbon. Guo et al. [4] experimentally researched the adsorbability of modified activated carbon for CO_2_. Their results showed that adsorption was optimal after chemical modification using a mixed liquor of KOH and ethanol. New types of porous carbon material, such as carbide, carbon molecular sieves, and activated carbon, have been produced via template synthesis and physical or chemical activation. Optimizing the pore structure improves diffusion and adsorption capacity and thus increases the maximum capture capacity of adsorbents per unit weight [5–8].

Lan et al. [9] used 3- to 5-year-old Moso bamboo (*Phyllostachys edulis*) to prepare porous charcoal. Carbonization temperatures of 400, 600, and 800°C were adopted in their work. They found that bamboo carbonized at 600°C had the best Brunauer-Emmett-Teller (BET) specific surface area (4.45 m^2^/g) and Langmuir surface area (183.39 m^2^/g). Wang et al. [10] investigated the effects of manufacturing conditions on the adsorption of heavy metal ions by Makino bamboo charcoal. They found that the specific surface area and iodine number of bamboo charcoal activated at 900°C were larger than those of bamboo charcoal activated at 800°C. Moreover, the specific surface area of bamboo charcoal activated at 800°C by CO_2_ was larger than that of charcoal activated by steam. Jiang [11] examined the factors that influence the bamboo pyrolysis process, such as the terminal pyrolysis temperature, carbonization speed, bamboo moisture content, and bamboo dimensions. Among these, the terminal carbonization temperature most significantly affected bamboo charcoal quality and properties. The adsorption capacity of bamboo charcoal for methanal, benzene, methylbenzene, ammonia, and chloroform was also determined. Horikawa et al. [12] used Moso bamboo as the raw material for preparing activated carbon by chemical activation with K_2_CO_3_ and physical activation with CO_2_. Their experimental results suggest that some of the prepared activated carbon have strong potential for application as adsorbents for adsorption heat pumps, desiccant humidity conditioners, and general humidity conditioning. Antal et al. [13] investigated the influences of substrate composition, heating rate, thermal pretreatment, final (peak) temperature, pressure, vapor-phase retention time, and catalyst type on the yield of charcoal from biomass. A significant increase in the yield was achieved with operation at elevated pressure in a stagnant gaseous environment. However, Mackay and Roberts [14] found that predictions of the carbon yield of a lignocellulosic material using a simple model that sums the yields of the substrate's cellulose, hemicellulose, and lignin components are misleading because the carbon yield is strongly dependent on the vapor-phase conditions present in the pyrolytic reactor. Asada et al. [1] studied the relationship between the carbonization temperature of bamboo charcoal made from Moso bamboo and the removal effect of harmful gases, including formaldehyde, toluene, benzene, ammonia, and odorants. They found that the benzene, toluene, indole, skatole, and nonenal removal effects were the highest for the bamboo charcoal carbonized at 1000°C and tended to increase with increasing carbonization temperature.

The present study investigates the CO_2_ capture capacity of Moso-bamboo-based porous charcoal. The main raw materials for making carbon-based porous adsorbents include wood and bamboo. As natural forests are crucial to soil and water conservation, most countries have forbidden their logging. Bamboo has high fiber density and a tough texture. Used as a raw material, bamboo can produce porous charcoal with a high specific surface area and high porosity. Bamboo grows fast and can be harvested every four years. Replacing wood with bamboo for the preparation of porous charcoal can reduce carbon dioxide emissions. There are over 1000 species of bamboo [15]. Moso bamboo is a temperate species of giant timber bamboo native to China and Taiwan [15]. Moso bamboo possesses a remarkable cellulose structure, making it a promising microporous material with excellent CO_2_ adsorption capacity. It is thus used to make charcoal in the present work.

## 2. Experimental Method

### 2.1. Sample Preparation

**Figure 1:**
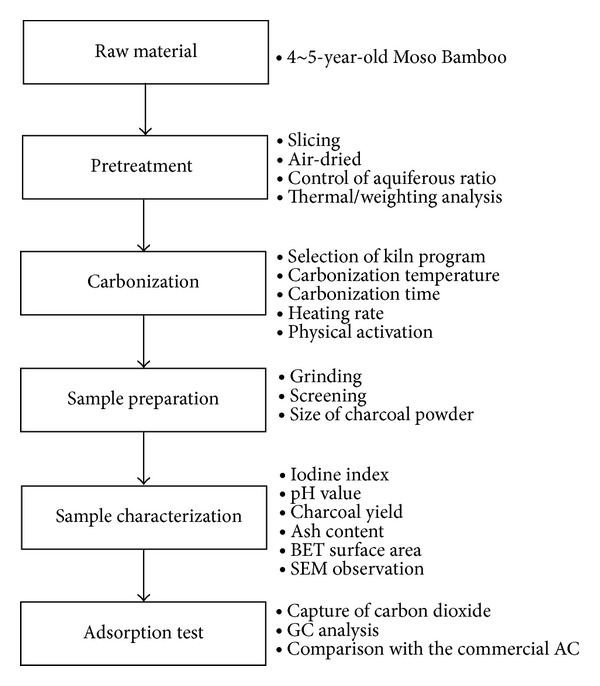
Procedure for preparing the Moso-bamboo-based porous charcoal.

Indigenous Moso bamboo, 4~5 years old, was used for preparing porous charcoal. The moisture content of raw bamboo directly influences the pyrolysis time and the consumption of heating energy. Cracks easily form in high-moisture-content bamboo due to nonuniform heating in the charcoal kiln, degrading the quality of the obtained charcoal. The bamboo specimens were thus air-dried before carbonization. The moisture content in the bamboo specimens was controlled to be in the range of 13~16%. The air-dried bamboo was sliced into strips with dimensions of 12 cm (length) × 4 cm (width) × 2~3 cm (thickness). Specimens of Moso bamboo were carbonized in a nitrogen ambient at kiln temperatures in the range of 600 to 1000°C. The carbonized bamboo charcoal was ground into powder with 60, 100, and 170 meshes, respectively, using a mechanical grinder. Prior to measurements, the specimens were pretreated at high temperature in a flowing inertia gas in order to remove any contaminants. The adsorption capacity of the porous bamboo charcoal was compared to that of commercial activated carbon (350 mesh). The experimental flow chart is shown in Figure 1.

### 2.2. Reactors

A compact charcoal kiln (HP-Cube, President Honor Industries Co., Ltd, Taiwan) was used to produce bamboo charcoal. Its maximum capacity is 1 kg of raw bamboo per batch. Induction heaters, which incorporate a coil directly fed from the electricity supply, were installed in the kiln walls for generating heat energy to carbonize raw bamboo. Temperature sensors were placed inside the charcoal kiln for monitoring the carbonization temperature. The carbonization temperature is programmably controlled by setting the temperature of the bamboo pyrolysis process. The bamboo specimens were heated from room temperature to the carbonization temperature with nitrogen gas used as the carrier gas at a constant flow rate (300 mL/min). A higher heating rate may result in a lower charcoal yield [16]. The heating rate was thus set at an accepted range of 5~10°C/min. Carbonization temperatures of 600, 700, 800, 900, and 1000°C were adopted for comparing the quality of bamboo charcoal. After the set carbonization temperature was reached, the bamboo was carbonized for 120 min at that temperature. The heater and gas flow were then turned off and the carbonized bamboo charcoal was allowed to cool naturally inside the kiln to room temperature.

### 2.3. Determination of Charcoal Yield

The charcoal yield, *Y*_char_ (%), produced by a kiln is given by *Y*_char_ = *m*_char_/*m*_raw_, where *m*_char_ is the dry mass of charcoal taken from the kiln and *m*_raw_ is the dry mass of the raw bamboo loaded into the kiln.

### 2.4. Experimental Measurement of Specific Surface Area and Adsorption Isotherms of Nitrogen at 77.3 K

Surface area and porosity are two important physical properties that determine the quality and effectiveness of bamboo charcoal. Differences in the surface area and porosity of charcoal particles can greatly influence its adsorption performance and characteristics. ASAP 2010 (Accelerated Surface Area and Porosimetry System, Micromeritics), which comprises an analyzer equipped with two sample preparation ports and one analysis port, a control module, and an interface controller, provides high-quality surface area (BET) and porosity measurements for various types of porous material based on gas adsorption theory [17]. ASAP 2010 is thus adopted to determine the BET surface area and total pore volume in this work. The multipoint (12-points) BET method and Langmuir method were used to determine the specific surface area. Nitrogen gas (N_2_) was used to determine the adsorption isotherms. The adsorption isotherm of nitrogen is measured in the range of relative pressure (*p*/*p*_*0*_) from 0.05 to 0.3 at 77.3 K, where* p*_*0*_ is the saturated vapor pressure of the system. As required, the specimen was out-gassed at 353 K for 1 h before the measurements.

### 2.5. Measurement of Iodine Adsorption Value

The iodine value of the Moso bamboo charcoal was determined according to ASTM D4607-94 (2011) [18], which is based on a three-point adsorption isotherm. The iodine adsorbed per gram of bamboo charcoal at a residual iodine concentration of 0.02 N is reported as the iodine value; that is, iodine value (mg/g) = [*A* − (DF) · (*B*)·(*S*)]/*M*, where *A* and *B* are 12693.0 and 126.93, respectively, DF represents the dilution factor, *S* is the volume in mL of standard sodium thiosulphate solution, and *M* denotes the bamboo charcoal used (g) [18].

### 2.6. Measurement of Carbon Dioxide Adsorption

To measure the CO_2_ adsorption capacity of bamboo charcoal powder, a breather tank with an inlet valve and a one-way gas relief valve was used as a CO_2_ container. A breather tank containing a specific weight of charcoal powder was taken as the experimental group, and that without charcoal powder was the control group. The bamboo charcoal prepared at various carbonization temperatures with various powder sizes (60, 100, and 170 meshes) was placed in the tank for adsorption tests for 5 days. Gas chromatography (GC; Hitachi 263-30) was used to measure the remaining CO_2_ concentration inside the breather tank each day. Comparing the changes of CO_2_ concentration in each breather tank allowed the adsorption capacity of various types of bamboo charcoal to be evaluated. The adsorption capacity of bamboo charcoal was compared with that of commercial activated carbon.

### 2.7. Gas Chromatography

CO_2_ concentrations were assessed using a gas chromatograph (263-30, Hitachi Co., Japan) equipped with a thermal conductivity detector, a flame-ionization detector (FID), and a glass-lined splitter. Operating conditions were 2 m × 3 mm i.d. capillary column (Porapak T); carrier gas: helium (flow rate: 40.0 mL/min); split ratio: 1/10; injection temperature: 100°C; column temperature: 50°C. The data processor was Hitachi chromato-Integrator D-2000.

### 2.8. Scanning Electron Microscopy Observation

**Figure 2:**
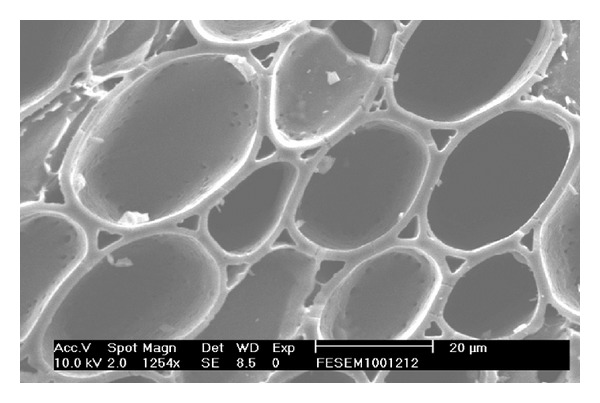
Cross-section SEM image of cavern pores in charcoal carbonized at 900°C.

Scanning electron microscopy (SEM; Philips, CM-200, Japan) was used to analyze the microporous structures of bamboo charcoal, including the cross-sectional parenchyma cells, thecal pores, and cell walls. SEM was operated at 10 kV in secondary electron imaging mode.  Figure 2 shows a high-resolution SEM image of a typical cavern pore structure for bamboo charcoal carbonized at 900°C. Some internal micropores can be observed on the surface of the cavern pore. The external cavern pores work as channels, where fluid molecules can pass through.

### 2.9. Determination of Ash Content and pH Value

The ash content of bamboo charcoal was determined according to CNS 5581 (1980) [19], which involved heating the carbonized charcoal in an open crucible (HP-Cube, Chuan Hua Precision Co., Germany) to 800 ± 25°C and holding at this temperature for 3 h. The material that remains in the crucible is defined as ash; that is, %  char  ash = 100 × *m*_ash_/*m*_char_, where *m*_char_ is the initial dry mass of charcoal and *m*_ash_ is the dry mass of ash that remains following combustion of the carbonized charcoal. In this work, the pH value was measured according to CNS 697 and CNS 698 (1965) [20] using a Eutech meter (pH 700, USA).

## 3. Results and Discussion

### 3.1. Microscopic Structure of Porous Charcoal

**Figure 3:**
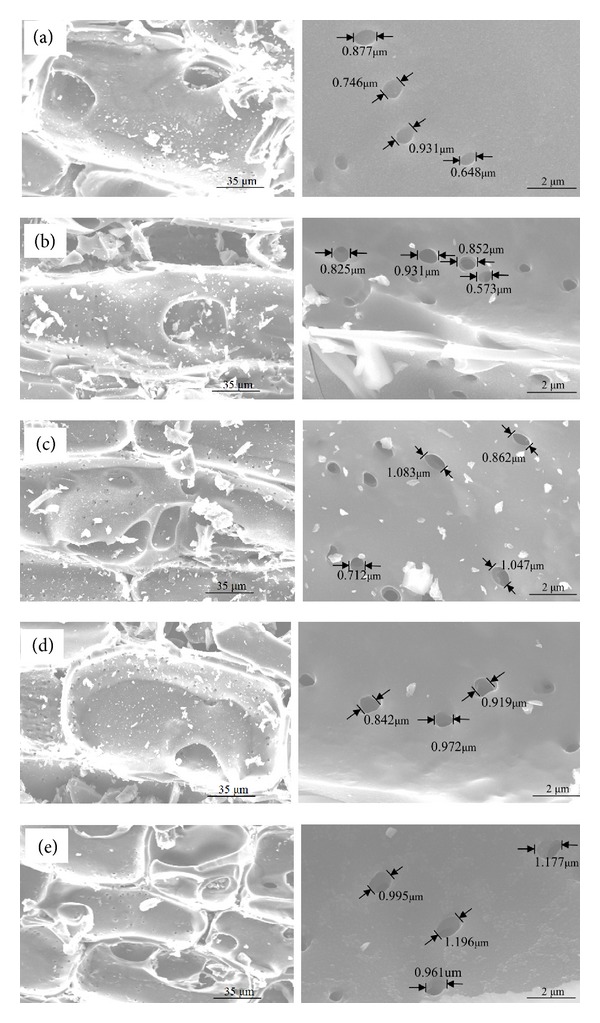
Thecal pore structure of charcoal carbonized at temperatures of (a) 600, (b) 700, (c) 800, (d) 900, and (e) 1000°C (left: radial section of thin-walled cells of charcoal, right: distributions of micropores in cavern cells).

The SEM microstructures of the thecal pores for porous bamboo charcoal prepared at carbonization temperatures of 600, 700, 800, 900, and 1000°C are shown in Figures 3(a)–3(e), respectively. As shown, the porous bamboo charcoal has a clear thecal pore structure. The thickness of the cell wall decreased with increasing carbonization temperature. According to Figures 3(a)–3(e), the carbonization at 600 and 1000°C resulted in the lowest and the highest thin-walled cell density, respectively. In addition, the thecal pores penetrated microcracks between the cell walls of thin-walled cells, as shown in Figure 3(e). This phenomenon is beneficial for expanding the carbon pore distribution and increasing the adsorption surface area. A comparison of the structures of cavern pores for samples carbonized at 600 to 1000°C indicated that carbonization at 800 and 1000°C produced the most micropores and the largest micropores, respectively, which is closely related to the adsorption capacity of porous charcoal.

### 3.2. Fundamental Properties of Moso-Bamboo-Based Porous Charcoal

**Figure 4:**
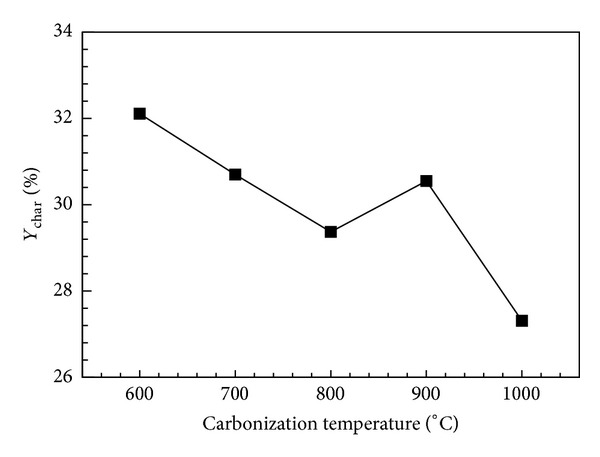
Charcoal yields for various carbonization temperatures.

**Figure 5:**
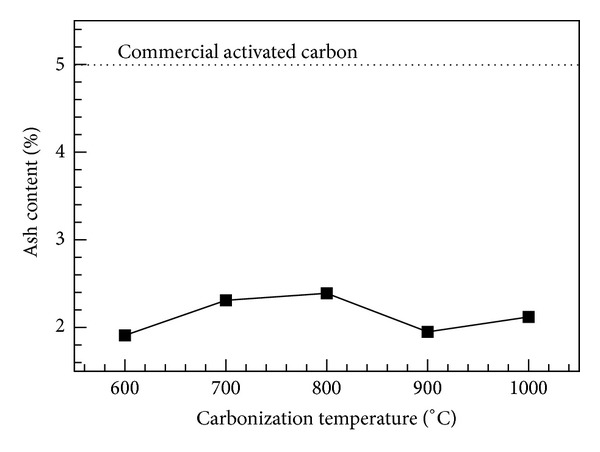
Ash content as a function of carbonization temperature.

This study investigated the effect of carbonization temperature (600, 700, 800, 900, and 1000°C) on the charcoal yield of Moso bamboo. The charcoal yield, *Y*_char_ (%), produced by a kiln is given by *Y*_char_ = *m*_char_/*m*_raw_, where *m*_char_ is the dry mass of charcoal taken from the kiln and *m*_raw_ is the dry mass of the raw bamboo loaded into the kiln. The process at each temperature was repeated six times to reduce experimental error.  Figure 4 shows the charcoal yield for bamboo carbonized at various temperatures. As shown, carbonization at 600 and 1000°C produced the highest (32.15%) and lowest (27.4%) average charcoal yields, respectively. The charcoal yield decreased with increasing carbonization temperature. The bamboo was burnt in a high-temperature calcinator to measure the ash content. The measured ash content values for bamboo charcoal calcined at various temperatures are shown in Figure 5. The carbides produced at different carbonization temperatures resulted in ash content values in the range of 1.91~2.39%. The ash content in inorganics is usually higher than that in wood coal [9]. The experimental results show that the ash content values are roughly independent of the carbonization temperature. Moreover, the ash content values of Moso bamboo are significantly lower than that of commercial activated carbon (~5.0%).

**Figure 6:**
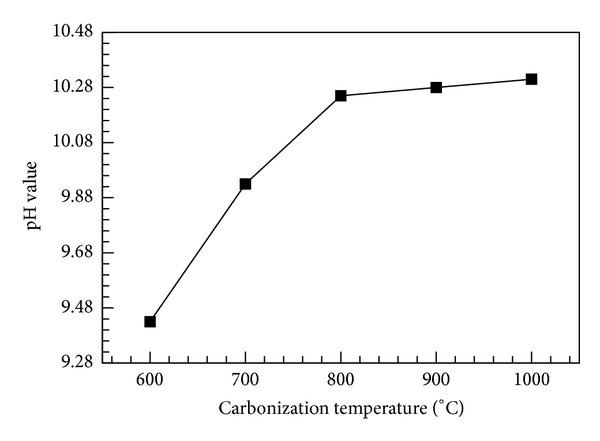
pH value versus carbonization temperature.

**Figure 7:**
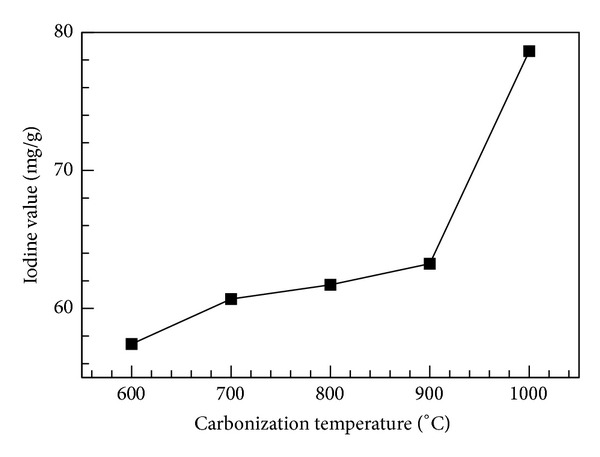
Iodine adsorption quantity as a function of carbonization temperature.

As the organic substances in bamboo were heated and decomposed, various pyrolysates formed and were discharged from the kiln, forming bamboo charcoal, which was rich in alkaline metals and mineral substances. Therefore, the measured pH values of bamboo charcoal inclined to have an alkaline reaction. As shown in Figure 6, a large change in pH value occurred at carbonization temperatures of 600~800°C, and a small change occurred for temperatures higher than 800°C. The highest pH value (10.33) was obtained for a carbonization temperature of 1000°C. The iodine number was used to represent the adsorption capacity of low-molecular-weight compounds. As shown in Figure 7, the iodine adsorption quantity gradually increased with increasing carbonization temperature in the range of 600~1000°C. The highest adsorption value (78.64 mg/g) was obtained at a carbonization temperature of 1000°C. At this temperature, micropores form in the thin-walled cells, increasing the adsorption pore surface area of the bamboo charcoal.

### 3.3. Relationship between Carbonization Conditions and BET Surface Area

**Figure 8:**
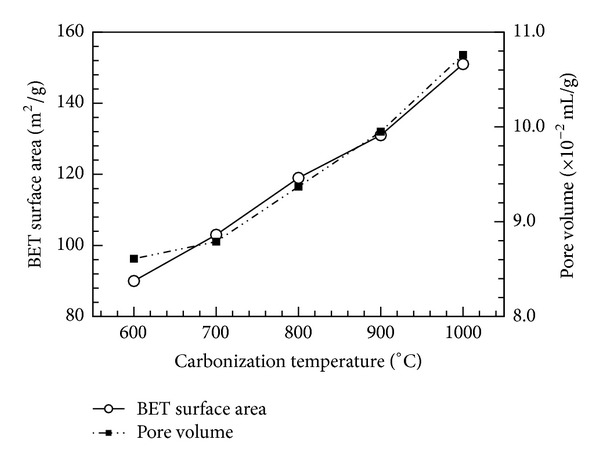
BET surface area and pore volume as functions of carbonization temperature.

**Figure 9:**
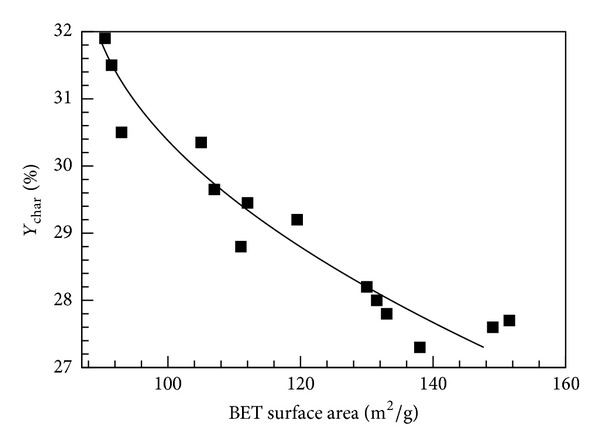
Charcoal yield as a function of BET surface area (solid line denotes fitted trend).

The pores in bamboo charcoal form during the carbonization process. Carbides with weak bonds are pyrolysed and chemically combined into small molecules and released, leaving behind caverns and micropores. Generally speaking, the specific surface area is regarded as a physical indicator of the adsorption capacity of an adsorbent. An increase in the specific surface area represents an increase in the number of adsorption sites in the adsorbent. ASAP 2010 was used to evaluate the BET surface area for porous bamboo charcoal carbonized at temperatures of 600~1000°C with a retention time of 120 min. Nitrogen was adopted as the replacement gas. When the van der Waals force of nitrogen reached its maximum in the liquid state, the nitrogen adsorption quantity at 12 relative pressures (*p*_*s*_/*p*_*o*_) at 77.3 K was tested. When the relative pressure was 0.05~0.3, the gas molecules formed an adsorption monolayer on solid surfaces. When the relative pressure was greater than 0.3, an adsorption multilayer formed, resulting in a detectable variance in measurements. According to the test results, various factors influence the BET area evaluation. As shown in Figure 8, the BET surface area and pore volume of bamboo charcoal increased with increasing carbonization temperature. The BET surface increments were 13.61 m^2^/g (600–700°C), 15.77 m^2^/g (700–800°C), 12.64 m^2^/g (800–900°C), and 20.35 m^2^/g (900–1000°C), with the highest increase from 900 to 1000°C. The BET surface area and pore volume reached their maximum at 1000°C. This can be ascribed to the significant amount of volatile matter released during carbonization. When Moso bamboo was carbonized at a lower temperature, the residues blocked the pores due to incomplete carbonization (i.e., tar formed). Tar is derived from the degradation of hemicellulose, cellulose, and lignin. It not only blocks the pore structure but also obstructs the formation and development of new pores, resulting in low total pore volume and low specific surface area. Although the BET surface area and the pore volume value increase with carbonization temperature, the overall charcoal yield decreases significantly, as shown in Figure 9. The weight loss of bamboo charcoal increases with increasing carbonization temperature, whereas the overall volumetric density decreases.

### 3.4. Relationship between Charcoal Powder Size and Adsorption Capacity

Adsorption is a consequence of surface molecular interaction based on the minimization of surface energy. Due to the bond deficiency of an adsorbent's surface atoms, it is energetically favorable for the atoms to bind to allochthonous molecules either by van der Waals forces (physisorption) or by chemical reactions (chemisorption). Since physisorption is a spontaneous thermodynamic process, negative free energy is required [21]. The free energy change, which combines the change of enthalpy and entropy, along with the temperature, can be used to determine whether a process is spontaneous [22–24]. This mechanism can be summarized by the Gibbs free energy equation; that is, Δ*G* = Δ*H* − *T*Δ*S*, where Δ*G*, Δ*H*, and Δ*S* are the changes in free energy, enthalpy, and entropy, respectively, and *T* is the absolute temperature in Kelvin [21]. Since translational degrees of freedom of the gas molecules decrease upon adsorption onto charcoal, Δ*S* is negative for the process. Δ*H* must thus be exothermic for physisorption [21]. In chemisorption, the charcoals trap gas molecules via the formation of chemical bonds with the surface. This interaction is much stronger than physisorption and may be stronger than the bonds internal to the gas molecules, which can result in the dissociation of the gas molecule upon adsorption [21–24]. In the present work, the process of CO_2_ adsorption can be described by four stages [25]: (i) isolated sites on the charcoal surface start to adsorb CO_2_ molecules at a low gas pressure; (ii) coverage of CO_2_ molecules increases with increasing pressure, forming an adsorption monolayer; the Langmuir model [26] can be used to calculate the surface area of bamboo charcoal; (iii) further increasing the gas pressure initiates multilayer stacking, and some smaller pores on the charcoal surface are filled; the BET formulation [27] can be used to evaluate the bamboo charcoal's surface area; (iv) with further increases in the gas pressure, the charcoal surface becomes entirely covered and all the pores become filled; the pore diameter, volume, and distributions can be determined using the Barrett-Joyner-Halenda calculation [28].

**Figure 10:**
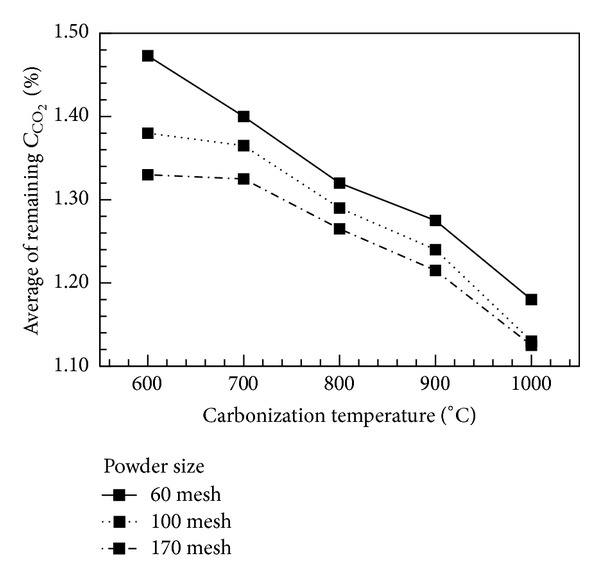
Average concentration of remaining CO_2_ as a function of carbonization temperature for charcoal with various powder sizes.

**Figure 11:**
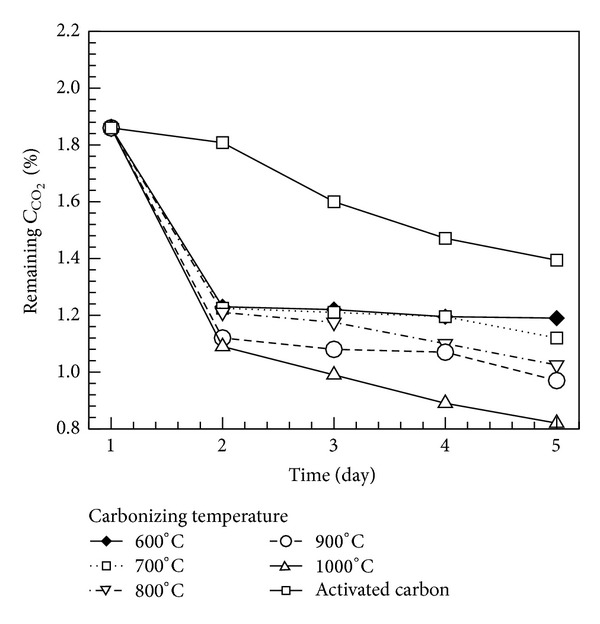
Remaining concentration of CO_2_ as a function of adsorption time for porous charcoal carbonized at various temperatures.

In this work, the size effect of bamboo charcoal powder on the adsorption of CO_2_ was investigated. The original CO_2_ concentration in the breather tank was 1.854%. Bamboo charcoal carbonized at various temperatures with various powder sizes (60, 100, and 170 meshes) was placed in the tank for adsorption tests for 5 days. The remaining CO_2_ concentration, as shown in Figure 10, denotes the average concentration value for the five days. Regarding the effect of powder size on CO_2_ adsorption, the results show that the powder size of 170 mesh has the best adsorption capacity. The adsorption capacity increases with increasing carbonization temperature. The remaining CO_2_ concentration reaches its lowest level for bamboo charcoal carbonized at 1000°C with a size of 170 mesh.  Figure 11 shows the capacity of CO_2_ adsorption for the charcoal powder carbonized at various temperatures with a powder size of 170 mesh. After adsorption for 5 days, the concentration of CO_2_ in the breathing cylinder significantly decreased from 1.854% to 1.169, 1.099, 1.012, 0.946, and 0.836% for bamboo charcoal carbonized at 600, 700, 800, 900, and 1000°C, respectively. The daily increase in the adsorption effect of bamboo charcoal carbonized at 1000°C is much higher than those for other temperatures, and the difference in adsorption capacity increases daily. Thus, bamboo charcoal carbonized at 1000°C with a size of 170 mesh has the best effect in CO_2_ adsorption.

This study compared the CO_2_ adsorption capacity of Moso bamboo charcoal and reagent activated carbon with a size of 350 mesh. The results are shown in Figure 11. On day 2, the CO_2_ concentration decreased by only 0.053% for the activated carbon, whereas the bamboo charcoal decreased the CO_2_ concentration by over 0.775%. However, the CO_2_ concentration decreased by only about 0.1% per day from day 3 to day 5. The remaining CO_2_ concentrations in the breathing cylinder after 5 days of adsorption were 0.836 and 1.394% for the bamboo charcoal powder and the activated carbon, respectively. The experimental results show that the adsorption capacity of bamboo charcoal powder is much better than that of reagent activated carbon.

## 4. Conclusion

The fundamental properties of Moso bamboo charcoal, including charcoal yield, ash content, pH value, BET surface area, iodine value, and pore volume, were experimentally investigated. The effects of carbonization temperature and powder size were discussed. The pore volumes tended to increase with increasing carbonization temperature. Moreover, the pore volumes were in the range of 8.6~10.7 cm^3^/g at carbonization temperatures of 600~1000°C. These results agree very well with those reported by Asada et al. [1]. Moreover, the charcoal produced at various carbonization temperatures had ash content ranging from 1.91 to 2.39%. The measured pH values of bamboo charcoal demonstrated to have an alkaline reaction and increased with increasing carbonization temperatures. The values agree very well with those reported by Lan et al. [9]. The BET surface area and pore volume were highest for the bamboo carbonized at 1000°C, with values of 151 m^2^/g and 10.76 × 10^-2 ^mL/g, respectively. The BET surface area reported in this work is much higher than that (4.45 m^−2^/g) reported by Lan et al. [9]. This can be ascribed to their work adopting a lower carbonization temperature (600°C) and a lower nitrogen gas flow rate (200 mL/min). Moreover, the use of a low carbonization temperature can result in residue blocking the micropores of charcoal due to incomplete carbonization and significant volatile matter release, such as tar. Experimental results show that porous bamboo charcoal carbonized at 1000°C for 2 h with a size of 170 mesh had the best effect on the adsorption of carbon dioxide.
